# A Simple and Light Weight External Fixator for Distraction Advancement Manoplasty

**DOI:** 10.5812/traumamon.6705

**Published:** 2012-10-10

**Authors:** Shahram Nazerani, Mohammad Hosein Kalantar Motamedi, Adel Ebrahimpoor, Jalal Vahedian, Tara Nazerani, Tina Nazerani, Bardia Bidarmaghz

**Affiliations:** 1 Department of Surgery, Tehran University of Medical Sciences, Tehran, IR Iran; 2 Trauma Research Center, Baqiyatallah University of Medical Sciences, Tehran, IR Iran; 3 Department of Orthopedics, Shahid Beheshti University of Medical Sciences, Tehran, IR Iran

**Keywords:** External Fixators, Osteogenesis, Distraction

## Abstract

**Background:**

With the growing interest in long bone distraction several types of distractors have been introduced; all have the same principle of an outer structure which acts like a scaffold and the distracting mechanism is a separate device which is mounted on this outer structure.

**Objectives:**

We have used a simple and very light weight external fixator we designed and discuss the results of distraction and advantages of this device .

**Materials and Methods:**

We applied our distractor to treat 14 men and four women, with a mean age of 39 years. There were three thumbs and 23 fingers; 26 digits (18 patients) lengthened by distraction callotasis and second stage bone grafting evaluated accordingly.

**Results:**

All patients but one were satisfied with the results and a stable pinch and grip was obtained. After lengthening, all patients maintained sensation of the finger pulp, as assessed by the Semes - Weinstein test.

**Conclusions:**

The superiority of this device for manoplasty lies in its simplicity of construction in addition to being, lightweight and also eases of application.

## 1. Background 

From the first reports of a new technique, i.e. long bone distraction, from Russia by Dr. Ilizarov there has been growing interest in this method and several types of distractors have been introduced (1-3).They all have the same principle of an outer structure which acts like a scaffold and the distracting mechanism is mounted on this outer structure and the distraction mechanism slides over this outer structure or the scaffold.

## 2. Objectives

**Figure 1:**
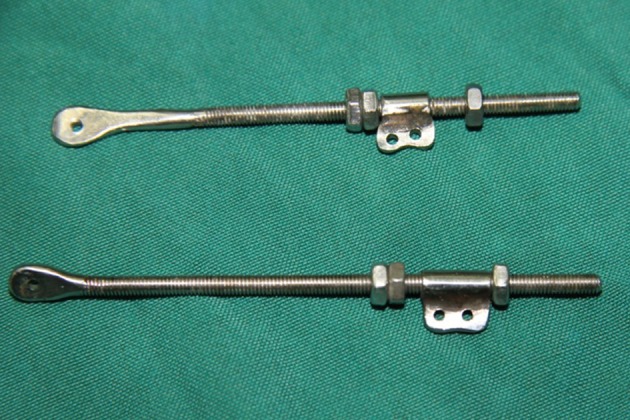
The Rods Can be of Differing Lengths for Fingers or in Children who Have Smaller Phalanges

**Figure 2:**
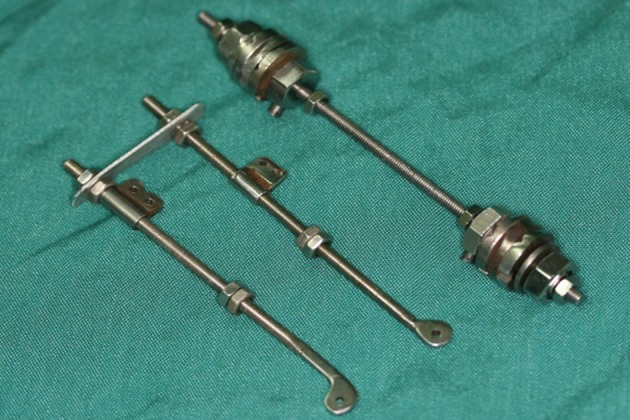
Ilizarov Original Device and our Device Depicted Together; the Assembled Ilizarov Device Weighs 32.5 Grams and our Assembled Device Weighs 6.5 Grams

**Figure 3:**
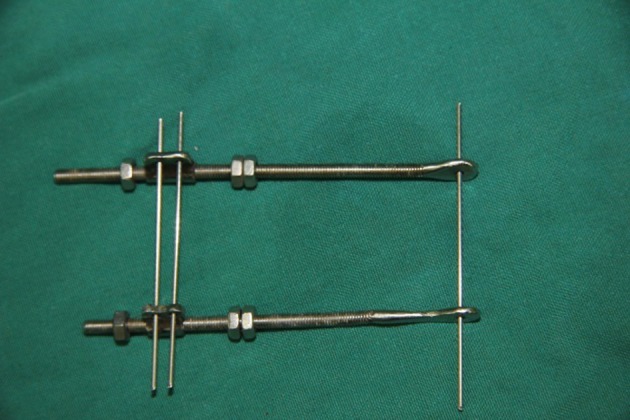
The Assembled Device is Depicted; the Distal Two Pins Transport the Bone Distally

The objective of this study was to test the effectiveness of the distractor we have designed; it is radically different from the current types because the distracting device is the outer structure (scaffold) or the "rods" themselves; the rods in currently available distractors act as only scafold and the distracting device is mounted over these rods. In our device the rods are long screws with an "eye" at one end and the sliding sleeve with two "eyes" for the pins which will distract the bone and these sleeves slide over the rods by tightening the bolts behind the sleeve on the rods (Figures 1, 2, 3).

## 3. Materials and Methods

Between 2007 and 2012, we carried out distraction callotasis on 45 digits in 32 patients. In order to consider the factors which influenced the period of healing, we excluded children from the study. These left 26 digits in 18 patients. There were 14 men and four women, with a mean age of 39 years (18 to 62). All the digits had had traumatic amputation. Assembling of the distractor is very simple and consists of insertion of one proximal pin at the most proximal part of the bone to be distracted or sometimes in the metacarpal proximal to the bone to be distracted and two parallel pins at the distal part of the bone to be distracted. For the metacarpal distraction one pin is inserted longitudinally in the distal part of bone to be distracted and the proximal part of pin is bent as a hook which will grasp the bone and exert traction.

**Figure 4:**
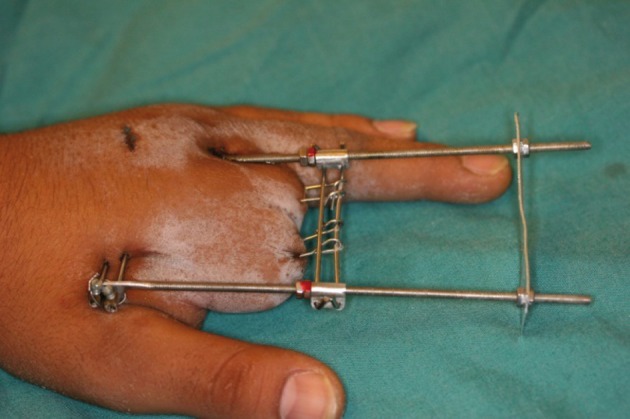
The Assembled Device is Seen with the Distal Balancing Plate to hold the Device in a Complete Rectangle

After assembling the device with a distal balancing sheet plate to hold the configuration of the distractor in a perfect rectangle the osteotomy is performed and the wound is closed (Figure 4). The distraction begins after three or four days depending on the wound condition and propelled forward till the desired length is achieved. In this series the lengthened bones involved three thumbs (2 metacarpals and 1 distal phalanx), nine index fingers (1 metacarpal and 8 proximal phalanges), seven middle fingers (2 metacarpals, 4 proximal phalanges and 1 middle phalanx) and seven ring fingers (4 proximal phalanges and 2 middle phalanges and 1 distal phalanx). The level of the osteotomy was at the proximal metaphysis in 10 digits (3 metacarpal and 7 proximal phalanges), at the middle diaphysis in 13 digits (7 metacarpals, 5 proximal phalanges and 1 middle phalanx) and at the distal metaphysis in three digits (3 proximal phalanges). The external fixator which we have designed is a simple device consisting of two threaded rods or long screws that are joined by pins passing through the bone and the distraction part of the device is a steel sleeve that has eyes for the pins and slides easily over the rods. Two screws behind the sleeve act as the distraction mechanism and two bolts are necessary to hold the distractor, the second one behind the first bolt acts as a "brake" to prevent back spiraling of the first bolt with increasing pressure of distraction. To hold the fixator in a perfect rectangle a steel sheet is inserted at the distal part of the fixator to hold it straight.

## 4. Results

Minor pin-track infection occurred in two digits, but this healed after local treatment. During lengthening of the thumb, an adduction contracture occurred in one metacarpal lengthening and a flexion contracture of the metacarpophalangeal joint occurred in another patient. In the other digits, a flexion contracture of the metacarpophalangeal joint occurred in 5 digits (1 metacarpal and 4 proximal phalangeal lengthening). An extension contracture of the proximal interphalangeal joint occurred in two cases of proximal phalangeal lengthening. In the remaining patients, active exercises and splint therapy reduced these contractures, although a flexion contracture of the metacarpophalangeal joint of 20° remained after two proximal phalangeal lengthenings.

The clinical results were assessed to determine whether the expected length had been achieved, and the external fixation index (EFI) and the healing index (HI) were determined. The EFI is the time needed for an external fixator to provide 1 cm of lengthening. The HI is the time taken to achieve consolidation in the gap for 1 cm of lengthening. There was no significant correlation between the HI and the age of the patient. There was no significant relationship between the distraction length and the HI or the EFI. The site of the osteotomy did not significantly affect the HI (P = 0.0974).

### 4.1. Statistical Analysis

Comparison of the HI for patients with and without complications was assessed by the Mann-Whitney U test and that of the HI between osteotomy levels was by the Kruskal-Wallis test. Differences were considered to be significant when the P value was less than 0.05. The HI with our device and the reported HI by other devices was identical although we adopted the bone graft protocol and when we reached the desired length we stopped the distraction and inserted a bone graft in the defect. In two 18-year-old patients who had lengthening of the proximal phalanges and one 25-year old patient with lengthening of the middle phalanx, the operations were performed for cosmetic rather than functional improvement. In the remaining 15 patients, surgery was undertaken in order to improve pinch function. Of these, all but one was satisfied with the result and a stable pinch grip was obtained. After lengthening, all patients maintained sensation of the finger pulp, as assessed by the Semes- Weinstein test. All but one patient, the 25-year-old with lengthening of the middle phalanx, indicated that they would accept the same procedure if they had the same problem again

### 4.2. Case Presentation

**Figure 5:**
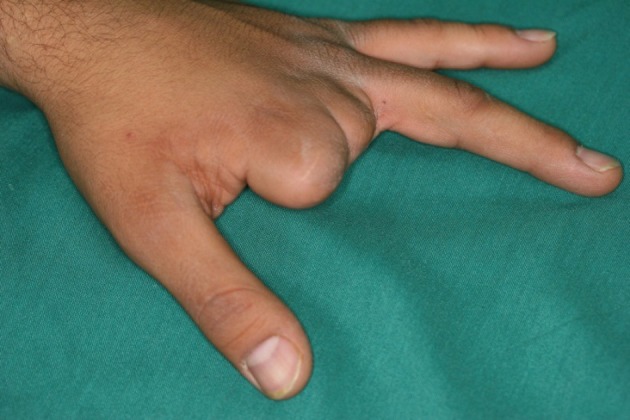
The Hand in Dorsal View

**Figure 6:**
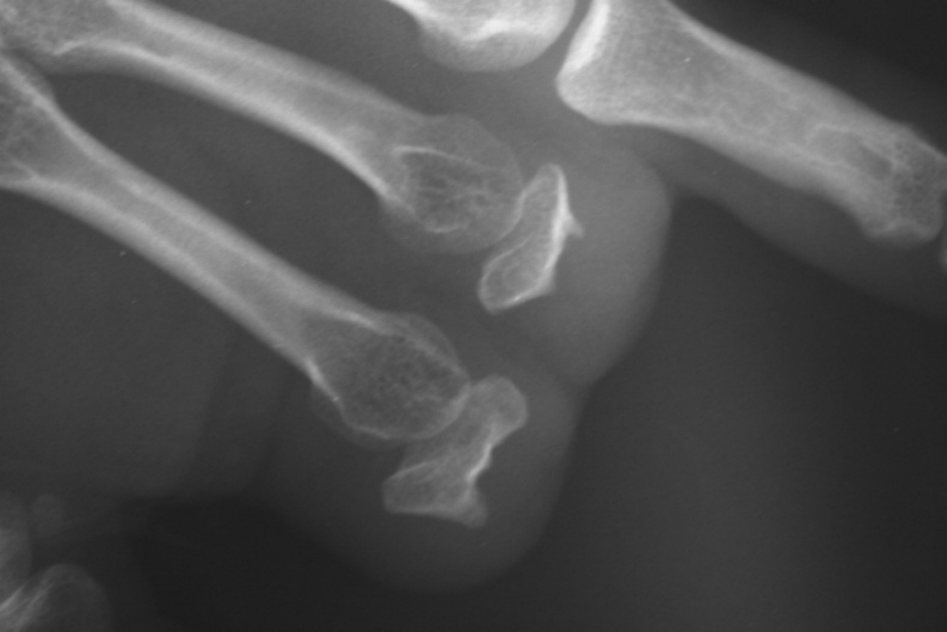
The X Ray of the Remaining Phalanges

**Figure 7:**
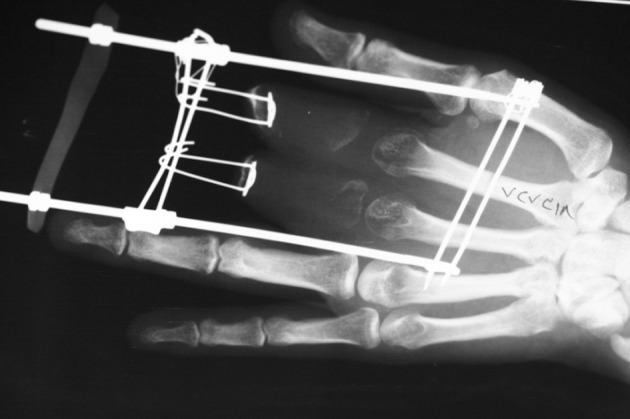
The Distraction is Complete

**Figure 8:**
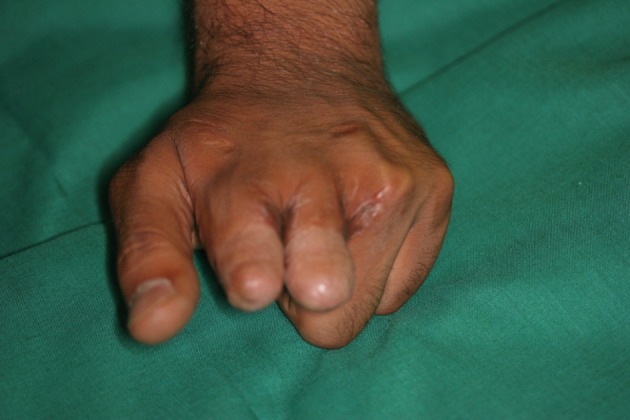
The Bone Graft and Web Deepening Procedure has been done

**Figure 9:**
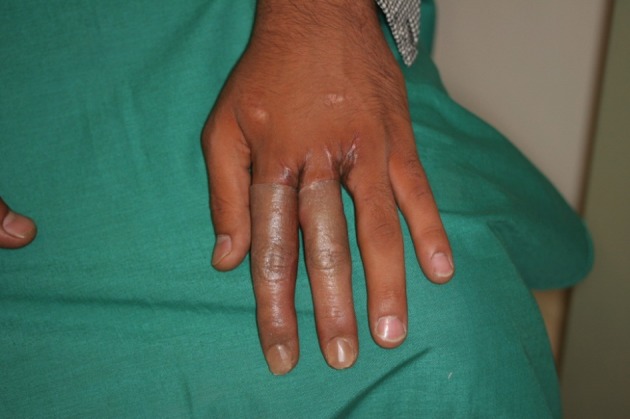
The Patient with Finger Prostheses in Place

A 26 year old Taxi driver with amputation of index and long fingers from a childhood accident requested lengthening of the stumps so he can wear finger prostheses (Figures 5 and 6). The remaining phalanges were too short for distraction with two parallel pins so we inserted a small two holes plate at the osteotomy site in order to get more anchorage for distraction. The distraction was successful and a length of 24 mm was achieved which was bone grafted at the second stage (Figures 7 and 8). The graft healed but with some volar bending of the proximal bones. After a web deepening procedure with skin graft he was able to wear the finger prostheses he had requested (Figure 9).

## 5. Discussion

In our study here were 14 men and four women, with a mean age of 39 years. There were three thumbs and 23 fingers. The distractor we designed is very lightweight (6.5 grams in a steel alloy model) and compared to other devices on the market such as the Ilizarov Device with 32.5 grams weight, has several advantages such as uniform bilateral distraction and slim design in addition to being compact and lightweight. The distractor consists of rods which are long screws and the distractor part is a steel tube sleeve which is propelled forward by tightening the bolts under it. The time to complete distraction and long term results was identical to relevant published literature. Distraction lengthening in the hand has become an established option for reconstruction in congenital anomalies and after traumatic amputations (4, 5). In 1979, Matev first reported gradual distraction osteogenesis of digits (4). There are several advantages and disadvantages of callotasis in the hand. It is less invasive than other techniques since gradual distraction is possible, exercise can be carried out during treatment and sensation is maintained. Disadvantages include longer treatment times with an associated higher rate of complications and a need for complicated and bulky instrumentation. Pensler described numerous complications which may occur during the course of distraction. Pensler concluded that distraction osteogenesis of the digits was superior to other methods and offered safe, reliable and predictable results(6).In children, good results without complications have been achieved with callotasis regardless of the original pathology or the lengthening required(7, 8).

In adults, complications such as fracture of callus or poor consolidation are more common. Matev recommended that a gap of 3 cm or more in patients over 20 years of age should be filled with bone graft without delay because spontaneous consolidation is doubtful(4, 9, 10). Most authors have followed this procedure, which entails gradual lengthening and bone grafting(2, 11-14). In our study, the mean HI was 101 days/cm (58 to 216) in the proximal phalanx. These data show that prolonged periods are needed for callotasis in adults. Aronson and Shen stated that the formation of newbone and mineralization in the experimental healing of distraction osteogenesis was better and quicker in metaphyseal than in diaphyseal bone(15). Our study supports those of other authors who have advocated the metaphyseal site for the osteotomy during distraction osteogenesis.

In this study, a simple and light weight distracter is introduced which is completely capable of acquiring the same results of the more expensive and sophisticated distracters available on the market. These distractors are small and light weight and are ideal for small children and the other noteworthy advantage is that they are inexpensive to manufacture , require no sophisticated machinery to construct and yield the same results.
